# Business economics in a pandemic world: how a virus changed our economic life

**DOI:** 10.1007/s11573-023-01135-x

**Published:** 2023-01-13

**Authors:** Wolfgang Breuer, Jannis Bischof, Oliver Fabel, Christian Hofmann, Jochen Hundsdoerfer, Tim Weitzel

**Affiliations:** 1 Department of Finance, RWTH Aachen University, Aachen, Germany; 2 Area Accounting & Taxation, University of Mannheim, Mannheim, Germany; 3 Department of Business Decisions & Analytics, University of Vienna, Vienna, Austria; 4 LMU Munich School of Management, Munich, Germany; 5 Department of Finance, Accounting & Taxation, Freie Universität Berlin, Berlin, Germany; 6 Otto-Friedrich-Universität Bamberg, Bamberg, Germany

**Keywords:** G00, H00, M00, O00

## What is special about the COVID-19 pandemic?

**Fig. 1:**
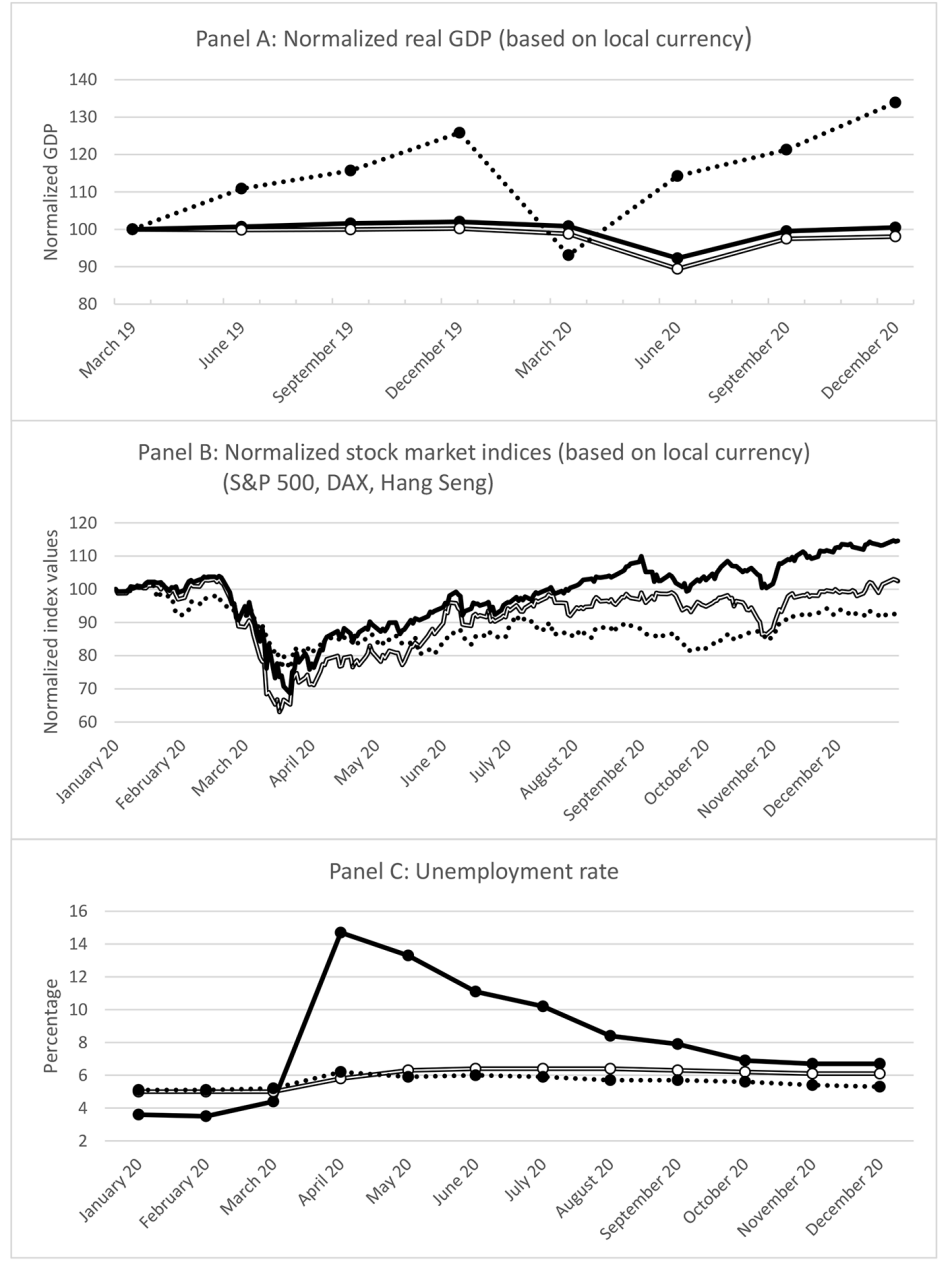
Development of key economic figures in China, Germany, and the US The solid lines represent US developments, while the double lines reflect the situation in Germany. The dotted lines depict the Chinese developments

Certainly, the SARS-CoV-2 virus causing COVID-19 was not the first microorganism haunting mankind and it will not be the last. However, in contrast to most other diseases in the twentieth and twenty-first century, this one had tremendous economic consequences even in the Western world. The large economic impact of the outbreak of the disease was not mainly caused by the lethality of the virus which is only of a moderate level, but in particular by the rigorous measures which were taken by governments to protect its peoples. Curfews and “lockdowns”, previously only known from war times, paralyzed for weeks and months the economic and cultural life in many countries particularly in Europe and Asia. Some say that this harsh reaction reflects a general shift in the mindset of many societies: the willingness for endurance and suffering has declined rapidly for the last few decades, leading also to pronounced developments in other fields of our societies. Be that as it may, in any case the beginning of the pandemic in spring 2020 coincided with dramatic downturns on the stock market and the labor market as well as severe reductions in economic output (see Fig. 1).

However, these severe negative developments turned out to be only transitory to a great deal and most economies recovered rather quickly. Certainly, this was to a large part owed to the success in research. In record time, researchers were able to first identify the cause of the new disease and then eventually to develop powerful vaccines against the virus. This worldwide concentration on COVID-19 related research is also reflected in the impressing numbers of articles published on the topic. According to the Web of Science, for 2019, there are only 33 articles containing “COVID-19” in their title, their abstract or key words, but already 82,697 in 2020, almost doubling to 160,242 articles in 2021. Even until mid-September 2022 there have already been additional 99,935 published COVID-19 related papers in the Web of Science. Though most of these papers stem from medicine journals, these are not the only source for publications related to COVID-19. However, economics and management journals seem only just to start contributing to the discussion right now. To be more precise, when looking at the top 5 economic journals (the American Economic Review, Econometrica, the Quarterly Journal of Economics, the Journal of Political Economy, and the Review of Economic Studies), from 2020 to mid-2022 there have only been four articles related to “COVID” or this particular “pandemic” in their title, their abstract or their keywords. Referring to several renowned management journals (Academy of Management Journal, Strategic Management Journal, Academy of Management Review, Journal of Management, Management Science), we find a similar pattern: overall only five publications in 2002, ten in 2021 and twelve so far in 2022. Apparently, there still seems to be much room for expansion. Nevertheless, several (other) journals have already published special issues on this topic (see, e.g., the Journal of Economic Perspectives, 2022, Vol. 36 (2), Small Business Economics, 2022, Volume 58 (2), the Journal of Financial and Quantitative Analysis, 2021, Vol. 56 (7), and the Journal of Business Research, 2020, Vol. 117) and now, the Journal of Business Economics is doing the same.

What are the motives for analyzing the COVID-19 pandemic from an economic (and management) point of view? This question will be addressed in the following Sect. 2. Against this background, we introduce the eight papers of this special issue in Sect. 3. Section 4 concludes.

## Why it pays to examine COVID-19 from an economic point of view

First of all, it is necessary to understand the short-term economic implications of the COVID-19 pandemic to support decision-makers in companies and politics in similar future situations. In this context, it is also of special interest to find out which insights can be generalized to other kinds of crises and which are specific for the handling of diseases. The better we understand the mechanisms behind crises as well as the impact and effectiveness of countermeasures, the better we can react in comparable situations in the future.

Secondly, although a great deal of the economic consequences might only be of a transitory nature, there are certainly long-term implications as well. According to the Human Development Report (2021/2022), the Human Development Index value has declined since 2020, erasing the gains of the preceding five years leading to new challenges in the field of political economy. However, there are also permanent effects to be observed on the firm level. For example, in human resource management, working from home has become an issue which cannot be ignored even after the end of the current pandemic due to technological innovations boosted in the wake of the disease and changes in human behavior. Something similar is true for the relevance of online shopping. Therefore, one important question is to distinguish between the transitory and the permanent effects of the pandemic. A second question is the handling of the permanent consequences in an effective way. A third question may be how to prepare for future crises of such a dimension.

The contributions of this special issue cover a broad range of sub-disciplines in business economics such as finance, human resource management, information systems, management accounting, and tax issues and aim at contributing to these main questions.

## The articles of this special issue

As already mentioned, governments worldwide reacted to the outbreak of the pandemic in an unprecedented way, and Christian Beer, Janine Maniora, and Christiane Pott now ask in their paper on the “COVID-19 pandemic and the capital markets: the role of government responses” the question which measures were most effective regarding improving investor sentiment and thus eliciting positive stock market reactions (Beer et al. 2023). In doing so, they want to understand what triggered investors’ optimism to regain so swiftly even in a situation where cases and deaths were still increasing. To this end, the authors distinguish between restrictive and supporting government policies. While the former ones may increase overall pessimism, the latter ones are particularly suited to reduce the feeling of uncertainty in the market and thus to raise trust among capital market participants leading eventually to stock market recovery.

Their empirical study examines the effect of the reactions of 180 governments on the connection between stock prices of S&P 500 firms and the changes in COVID-19 infections and deaths between January 1, 2020, and March 15, 2021. Governmental reactions were described by 16 indicators belonging to the three fields of containment and closure, the health system, and economic support, as recorded by Oxford University’s Government Response Tracker. Relying on country-specific revenues for firms it was possible to identify in detail consequences of governmental policies for firms.

Whereas public measures taken to contain the virus as well as economic support improve investor sentiment, governmental actions to strengthen a country’s health system aggravate the stock market situation. In addition, the firms that are highly exposed to the pandemic on the sales side profit in particular from public economic support, whereas the impact of containment and closure measures was not moderated in a similar way.

The authors’ findings help firms to anticipate the consequences of a pandemic and the corresponding measures taken by public authorities with respect to investor sentiment. Moreover, the paper may help governmental decision-makers to better assess the costs and benefits of potential countermeasures.

One quite specific instrument employed by governments to increase the resilience of firms is tax regulation, because taxes may generally act as an automatic stabilizer that mitigates the effects of economic crises on firms. The corporate tax may cushion firms from the influence of an economic downturn. In addition, short-term tax incentives can stimulate firm investment during crises. On the other hand, most corporate tax systems are designed asymmetrically, with an immediate and unlimited profit taxation, but a delayed and limited refund for tax losses. It is an open question how tax loss carryover regulations affect whether taxes are effective as automatic stabilizers. In their paper “Losses never sleep – the effect of tax loss offset on stock market returns during economic crises”, Reinald Koch, Svea Holtmann, and Henning Giese analyze whether more generous tax loss offset regulations are associated with a better stock price performance (weaker decline and stronger recovery of firm stock prices) during economic crises, i.e. the 2008 financial crisis and the COVID-19 crisis (Koch et al. 2023). They make the argument that firms will be provided with additional liquidity if loss carryback and, to a lesser extent, loss carryforward regulations are generous. This additional liquidity will reduce the bankruptcy risk and crisis-driven investment constraints.

The authors find that an unrestricted loss carryback and carryforward are associated with a weaker decline and more timely recovery of stock prices during the considered crises. As expected, the effect size is driven by the size of the tax rate. The positive effect of tax loss carrybacks is associated with pre-crisis profitability, as pre-crisis profits are a requisite for using tax loss carrybacks. If the reader interprets the findings as causal, the central message from this paper is that, in a crisis, extending tax loss offsets for firms may be a simple and effective policy measure to protect firms.

Another question which might be of interest for political decision-makers is the general reaction of firms and entrepreneurs in times of crises. In their paper on the “Socioeconomic status and entrepreneurial networking responses to the COVID-19 crisis”, Leif Brändle, Helen Signer, and Andreas Kuckertz take a network-oriented approach to examine whom entrepreneurs call in case of emergency and show that entrepreneurial reaction depends on the entrepreneur’s socioeconomic status (Brändle et al., 2023). Higher socioeconomic status leads to higher goal-oriented behavior. This means that other individuals are seen as instrumental, i.e., according to how useful they are for the entrepreneur under consideration. As a consequence, individuals of higher socioeconomic status may activate contacts to serve their needs and even replace contacts that are not deemed to be beneficial during the crisis. In contrast, entrepreneurs of lower socioeconomic status are more concerned with reciprocity on their network and try to avoid social risk. Therefore, in a crisis, these entrepreneurs may focus more on the needs of others in hope of future reciprocity offered when needed. Based on an experiment with a final sample of 122 participants, the authors find indeed evidence for their conjecture.

For firms it is not only essential to react ex post adequately to critical situations, but also to build ex ante capacities which help to become sufficiently resilient to external shocks like the outbreak of a pandemic. Moritz Sefried and Jan Riepe present an empirical study entitled “The benefits of banks’ IT investments in times of trouble. Evidence from loan loss accruals during the COVID-19 pandemic” which is based on 8,522 bank-quarter observations from 665 US banks to analyze how IT capabilities (proxied by the amount of IT investments) help to prepare for times of crisis (Sefried and Riepe 2023). In “normal” times, banks have quite a good understanding of the quality of their loan portfolio and thus can forecast their loan risk rather well based on past experience. However, this becomes harder or even impossible in situations with a structural break. The authors show that under such circumstances banks with higher IT investments are better able to estimate their loan loss accruals. From this finding, one may conclude that IT investments should become the more important the more volatile the banking environment gets.

The COVID-19 pandemic was associated with various social distancing measures at the individual, corporate, or government level, thus particularly affecting service industries and public administrations, where the presence of a customer, patient, or citizen is required for service provision to take place. In this respect, digitalized procedures likely improve the effectiveness of working from home for for-profit organizations, non-profit organizations, and public administrations. In their paper “The impact of digitalized communication on the effectiveness of local administrative authorities – Findings from central European countries in the COVID-19 Crisis”, Bernhard Hirsch, Fabienne-Sophie Schäfer, Aleksander Aristovnik, Polona Kovač, and Dejan Ravšelj examine how the use of digitalized communication tools affect the effectiveness of public service provision by various European local administrative units during the COVID-19 pandemic (Hirsch et al. 2023). Using survey data from local public managers in the Czech Republic, Germany, Poland, Slovenia, and Romania, the study finds that the COVID-19 pandemic accelerated the implementation of digitalized procedures in public administrations, and that digitalized procedures are key for effective local administrative units.

Even after restrictions had been relaxed to some degree, many firms chose to keep some of the working from home opportunities for their workforce as part of an effort to increase workplace flexibility (e.g., Barrero et al. 2021). While the increased flexibility through working from home offered some benefits for firms and helped increase their resilience during the crisis (Barry et al. 2022), the greater physical distance between the individual employees and their supervisors also posed a challenge to firms’ internal control systems, as it can lead to a lack of motivation and hardly observable misbehavior. In their paper on “Working from home and management controls”, Konstantin Flassak, Julia Haag, Christian Hofmann, Christopher Lechner, Nina Schwaiger, and Rafael Zacherl offer unique insights in how firms dealt with this challenge (Flassak et al. 2023). Their evidence comes from a survey among employees in a large international corporation. They show that working from home led to the organization relying more on standardization and employee participation in planning processes. While the organizational change resulted in more time that employees had to spend in meetings, it also came with some benefits such as an increased job focus. These findings contribute to current policy debates about workplace flexibility and help understand that physical distance between supervisors and employees does not necessarily harm the organization as a whole, at least if supervisors adjust internal communication and action controls correspondingly.

This raises the question of whether working from home is here to stay. Christian Kagerl and Julia Starzetz are able to draw on a large-scale and representative survey of German production sites in their paper “Working from home for good? Lessons learned from the COVID-19 pandemic and what this means for the future of work” (Kagerl and Starzetz 2023). Their investigation identifies the home office potential of the German private economy to comprise 25–30% of the workplaces. Also, since technical and IT issues appear to impose no problems any longer, especially larger firms intend to expand work from home even after the COVID-19 pandemic has come to an end. Predominantly, the site managements report positive effects of home office work, with the exceptions that they noted problems in communicating with their employees and when recruiting new staff.

The COVID-19 pandemic has clearly shown the value of information systems for rethinking and virtualizing work arrangements to counter the consequences of the virus (e.g., Hirsch et al. 2023, Kagerl and Starzetz 2023). But information systems have also been instrumental in fighting the virus itself. Prominently, digital contact tracing applications have helped in the pandemic response by collecting and using data on COVID infections. As a platform technology based on technical standards, such solutions are subject to network effects, i.e. the value of a solution increases with the number of its users. This, unfortunately, creates a variety of substantial coordination problems. In their paper “The dilemma of digital contact tracing: standardization on digital platforms under network effects“, Felix Büsching, Dennis Steininger, and Daniel Veit analyze how Google and Apple effectively set the standards that made a quick European solution possible but at the same time severely limited the individual leeway of other firms (Büsching et al. 2023).

The case offers a timely example of a classic battle of standards extended by the elements of a modern platform economy. The authors reveal competing concerns, such as fast diffusion and utility vs. privacy concerns, interdependent actors, such as small and large private firms and public authorities, and trade-offs regarding, among others, interoperability, digital sovereignty, privacy and security, technology performance, and market incentives.

Following the call to draw short- and long-term lessons from the pandemic, the authors disclose the complex multi-stakeholder decision context that was necessary to come up with a fast solution. They delineate the possible winners and losers in the short and the long run, and blueprint important elements of a strategy for how to make goal-oriented, well-informed decisions in the next crisis when, again, effective public-private coordination under time constraints might be needed.

## Conclusion

Overall, the papers from this special issue document the very unique challenges that many firms faced during the pandemic, often caused by tight governmental restrictions, but also frequently a consequence of a disruption in customer demand and supply chains. The breadth of the economic shock is unparalleled in recent history. However, the papers also show that some firms were able to successfully and efficiently respond to the economic challenges. Therefore, there is large heterogeneity in how hard the crisis has eventually hit the different firms. It is the responsibility of business research to examine the factors that explain the differences and that make firms resilient to the situation of an extreme crisis. The special issue contributes to this debate and the papers identify some of these factors such as the quality of a firm’s internal IT system and the level of digitalization or the flexibility of internal management controls.

Moreover, as a crisis is typically associated with organizational changes, there may also be some benefits of the COVID-19 pandemic as long, overdue adjustments were triggered by its outbreak. Examples relate to the widespread acceptance of working from home arrangements and the investment in digitalized communication tools in public administrations. In other words, benefits of the pandemic-induced changes manifest themselves as employees’ improved work-life balance and more effective public administrations. From an even broader perspective, it may be worthwhile to consider the COVID-19 pandemic through the lense of Schumpeter’s (2020) creative power of destruction. In this sense, even global crises also may open up windows of opportunity eventually creating new welfare. Hopefully, in hindsight, this will prove true for the COVID-19 pandemic as well.
